# Five- and Six-Membered Nitrogen-Containing Compounds as Selective Carbonic Anhydrase Activators

**DOI:** 10.3390/molecules22122178

**Published:** 2017-12-09

**Authors:** Adriano Mollica, Giorgia Macedonio, Azzurra Stefanucci, Simone Carradori, Atilla Akdemir, Andrea Angeli, Claudiu T. Supuran

**Affiliations:** 1 Department of Pharmacy, “G. d’Annunzio” University of Chieti-Pescara, Via dei Vestini 31, 66100 Chieti, Italy; giorgia.macedonio@unich.it (G.M.); a.stefanucci@unich.it (A.S.); simone.carradori@unich.it (S.C.); 2 Department of Pharmacology, Bezmialem Vakif University, Vatan Caddesi, 34093 Fatih/Istanbul, Turkey; aakdemir@bezmialem.edu.tr; 3 Neurofarba Department, Università degli Studi di Firenze, Via U. Schiff 6, 50019 Sesto Fiorentino (Florence), Italy; andrea.angeli@unifi.it (A.A.); claudiu.supuran@unifi.it (C.T.S.)

**Keywords:** carbonic anhydrase activators, diketopiperazine, ergothioneine, melatonin, spinacine

## Abstract

It has been proven that specific isoforms of human carbonic anhydrase (hCA) are able to fine-tune physiological pathways connected to signal processing, and that decreased CAs expression negatively influences cognition, leading to mental retardation, Alzheimer’s disease, and aging-related cognitive dysfunctions. For this reason, a small library of natural and synthetic nitrogen containing cyclic derivatives was assayed as activators of four human isoforms of carbonic anhydrase (hCA I, II, IV and VII). Most of the compounds activated hCA I, IV and VII in the micromolar range, with *K*_A_s ranging between 3.46 and 80.5 μM, whereas they were not active towards hCA II (*K*_A_s > 100 μM). Two natural compounds, namely l-(+)-ergothioneine (**1**) and melatonin (**2**), displayed *K*_A_s towards hCA VII in the nanomolar range after evaluation by a CO_2_ hydration method in vitro, showing a rather efficient and selective activation profile with respect to histamine, used as a reference compound. Corroborated with the above in vitro findings, a molecular modelling in silico approach has been performed to correlate these biological data, and to elucidate the binding interaction of these activators within the enzyme active site.

## 1. Introduction

Carbonic anhydrases (CAs, EC 4.2.1.1) are a large family of zinc-containing metalloenzymes found in prokaryotes and eukaryotes, involved in the reversible conversion of carbon dioxide into bicarbonate and a proton. The specific activation of this enzyme has been explored in animal models of neurodegenerative or age-related diseases with the aim of proposing new innovative pathways able to contrast cognitive impairment and brain functional loss [1,2,3,4]. As evidence of this concept, the management of such pathological conditions in which learning and memory are altered relies on a large plethora of studies demonstrating how neuronal responses, especially in the hippocampus, were strictly modulated by an increased bicarbonate conductance through synaptic GABA_A_ receptor channels and by extracellular signal-regulated kinase (ERK) phosphorylation in the amygdala [5,6]. Carbonic anhydrase could be genetically dysregulated in several deficiency syndromes, where mutations of CA-related genes led to clinical manifestations of autosomal recessive osteopetrosis, renal tubular acidosis, increased frequency of fractures and dental abnormalities, and delay of development [7]. Impaired neuronal functioning was often coupled to brain calcification affecting oligodendrocytes, astrocytes, and some pyramidal neurons [8]. Many isoforms of CAs are expressed in the central nervous system (CNS) contributing to signal processing, better performance in the Morris maze test, fear memory, attention gating of memory storage, spatial learning, cerebrospinal fluid composition and turnover, and long term synaptic transmission especially in the cortex and hippocampus [9,10]. Moreover, local pH fluctuations were shown to modulate physiological responses sustained by the NMDA receptors [11].

Up to date, CA activators are chemically represented by natural endogenous compounds (histamine, catecholamines, amino acids/peptides, carnosine) [12,13,14,15,16,17,18,19,20] and some synthetic derivatives (oligopeptides, pyridinium-azoles, selective serotonin reuptake inhibitors, histidine and histamine derivatives, imidazole/triazole/indazole/pyrazole/oxazole/thiadiazole derivatives) [21,22,23,24,25,26,27,28,29,30,31,32] presenting common features such as:

- Steric factors leading to a proper orientation for facilitating the proton shuttle from the entrance to the active site;
- Electronic factors like p*K*_a_ values in the range 6.5–8.0 for protonatable moieties suitable to establish a network of H-bonds with water molecules or amino acid residues;
- Structural characteristics like nitrogen-containing rings or chains to provide the protonatable moiety.

Collectively, this proposed pharmacophore would allow the binding of the activator to the entrance of the active site cavity differently from the classical inhibitors binding site, the establishment of a re-organization of H-bonds within this area, and the corresponding enzymatic activity facilitating the His64-mediated (His66 in CA VII according to the numbering of amino acid residues) proton shuttle transfer reaction.

The complete elucidation of this mechanism was carefully demonstrated by Temperini et al. with the support of in-depth kinetic, spectroscopic, and crystallographic studies [33]. For this reason, CA activators (CAAs) were widely designed as important pharmacological and diagnostic tools [34] showing CA activation profiles up to the nanomolar range, and with interesting isoform specificity [35,36].

The most promising results in terms of activation potency and isoform selectivity were disclosed in order to extrapolate the structure-activity relationship (SAR) useful for the design of novel CAAs. On the basis of the enzyme-activator adducts and keeping in mind the CNS distribution of hCA I, II, IV, and VII isozymes, we propose novel chemotypes incorporating all the above mentioned characteristics in five- or six-membered nitrogen containing structures to elicit this peculiar biological activity and to explore the chemical space around these positions.

**Figure 1:**
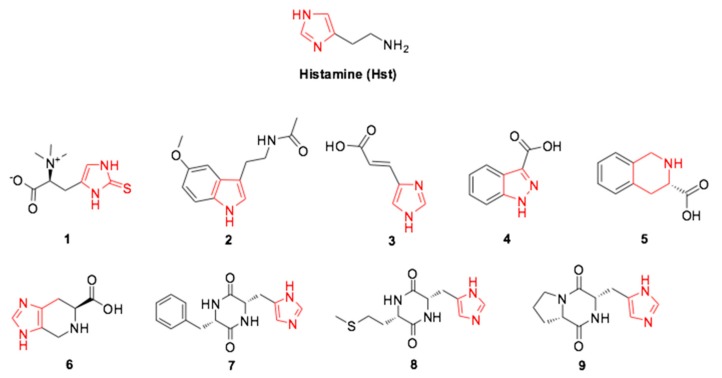
Chemical structures of histamine (Hst, reference drug), commercial products **1**–**6**, and synthetic compounds **7**–**9**.

We selected important derivatives such as l-(+)-ergothioneine (**1**), an anti-oxidant and chelating naturally-occurring compound containing a sulfur atom in the imidazole ring of histidine, melatonin (**2**), which is a first-line defense hormone against oxidative stress in the CNS, 4-imidazole-acrylic acid (**3**) and indazole-3-carboxylic acid (**4**), both incorporating acid and basic functions, TIC**^.^**HCl ((*S*)-1,2,3,4-tetrahydro-3-isoquinolinecarboxylic acid) (**5**) and spinacine ((*S*)-4,5,6,7-tetrahydro-1H-imidazo[4,5-*c*]pyridine-6-carboxylic acid) (**6**) in order to evaluate the impact of the presence/absence of an imidazole ring. Lastly, we synthesized and purified diketopiperazine derivatives (**7**–**9**) characterized by an imidazole ring linked to a nitrogen-containing six-membered nucleus (Figure 1).

All the compounds (**1**–**9**) were subjected to in vitro and in silico exhaustive evaluations as CA activators. This experimental design let us to discover innovative lead compounds not only selective and potent activators, but also endowed with appropriate physical-chemical characteristics such as balanced hydro/liposolubility and appropriate water solubility for the steps towards commercial drugs.

## 2. Chemistry

**Scheme 1:**
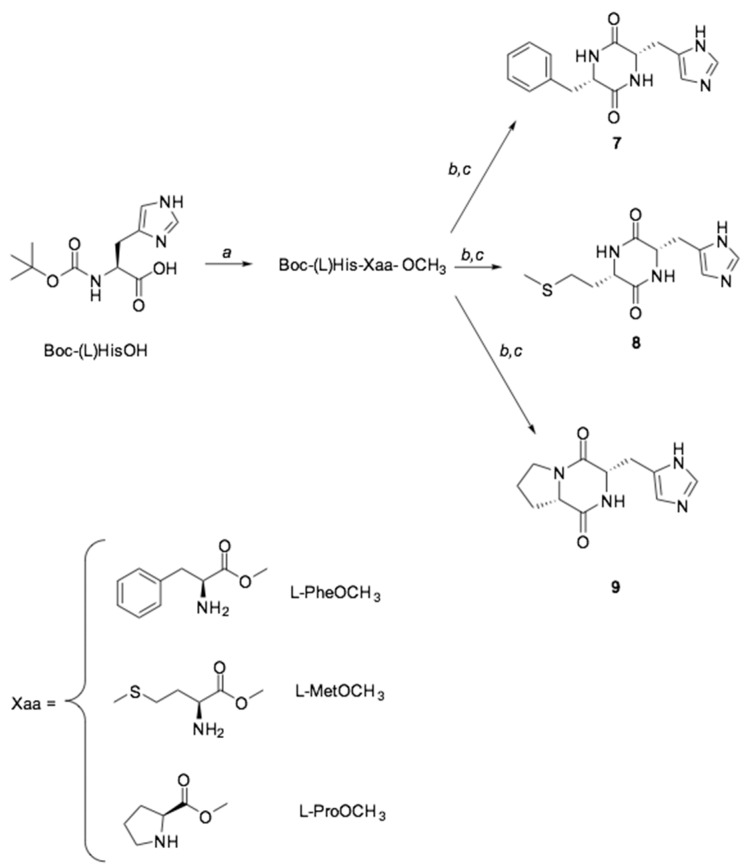
Synthesis of compounds **7**–**9**. Reagents and Conditions: (a) 1.1 equiv. DCC, 1.1 equiv. HOBt·H_2_O, 2.2 equiv. DIPEA, 1 equiv. L-PheOCH_3_ or L-MetOCH_3_ or L-ProOCH_3_, DMF, room temperature (r.t.) overnight; (b) TFA:DCM = 1:1 (*v*:*v*), r.t. 1 h; (c) 1.5 equiv. 0.1 M AcOH/2-butanol solution, 1 equiv. NMM, reflux overnight, 68% yield for **7**, 72% for **8**, 71% for **9**. For abbreviations, please refer to the Experimental Protocols section.

Derivatives **1**–**6** were commercially available. Conversely, a synthetic strategy to prepare derivatives (**7**–**9**) has been developed in our laboratory [37] involving Boc-solution chemistry (Scheme 1). The starting common dipeptide precursor was obtained by coupling Boc-(L)HisOH with L-PheOCH_3_, l-MetOCH_3_ and l-ProOCH_3_ in *N*,*N*-dimethylformamide (DMF). The obtained product was then deprotected and cyclized into the diketopiperazine derivatives (**7**–**9**) in high yield as reported in Scheme 1.

## 3. Biological Evaluation

All the synthesized and purchased (high purity grade) compounds were subjected to tests to measure their activation of the most important cytosolic isoforms, hCA I, II and VII, and the membrane-associated hCA IV by a stopped-flow, CO_2_ hydrase assay method. For comparison, we also discussed the activation profile of the standard histamine (Hst) against the same isoforms (Table 1).

## 4. Results and Discussion

Collectively, all compounds, except compound **2** (melatonin) have the possibility for accepting a proton, and can function as proton shuttles. From the data in Table 1, the following relationships between structure and activation profile can be concluded for this small library of nitrogen containing cyclic compounds and the first demonstrated standard activator (histamine):

(i) The abundant and cytosolic hCA I isozyme was moderately activated by compounds **3**–**9** similarly to histamine (micromolar range), whereas compounds **1** and **2** did not affect its enzymatic activity up to 100 μM;
(ii) As histamine, all compounds reported in this work were totally inactive against hCA II isozyme, usually abundant in the choroid plexus, oligodendrocytes, astrocytes, myelinated tracts, and myelin sheets;
(iii) With regards to the membrane-bound hCA IV isoform located on the luminal surface of cerebral capillaries and within the cortex, the hippocampus and thalamus, compounds **2**–**9** were poor activators (*K*_A_s = 53.4–80.5 μM) and with the same activation profile of histamine in the micromolar range. Conversely, compound **1** was totally inactive (*K*_A_ > 100 μM);
(iv) The last cytosolic hCA VII isoform displayed the most promising results in terms of activation and selectivity. Derivatives **3**–**9** activated this isozyme in the micromolar range, but compounds **1** and **2** were demonstrated to be rather efficient activators with *K*_A_ values of 820 and 120 nM, respectively (best-in-class activators, more selective up to two orders of magnitude). Compound **1** showed a very selective activation of only this isoform;
(v) Collectively, the synthetic diketopiperazines (**7**–**9**) were as potent micromolar activators as histamine.

The activation of the brain-associated hCA VII isoform is of particular interest not only because it is present in high levels in the cortex, thalamus and hippocampus, but also owing to the discovery of its pharmacological interaction with anti-neuropathic and anti-epilepsy agents.

## 5. Docking Studies

**Figure 2:**
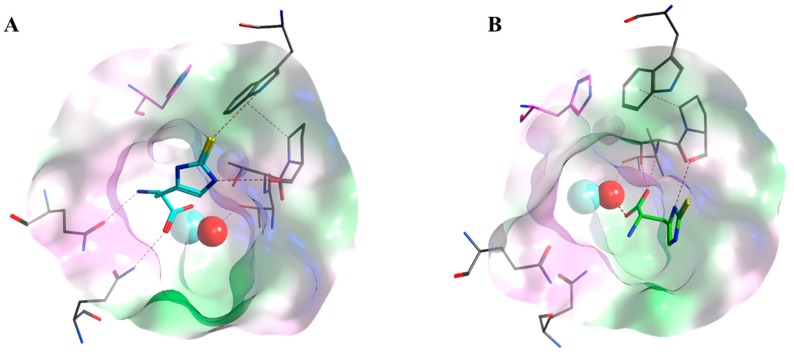
Two different docked poses (**A**,**B**) of l-(+)-ergothioneine (**1**) in the active site of hCA VII. The proton shuttle His66 is shown in magenta.

Compounds **1**–**9** and histamine were docked into the active site of hCA VII, the enzyme against which the ligands show the lowest measured *K*_A_ values, using the GOLD docking software and the ChemScore scoring function. In our previous studies, the ChemScore scoring function showed better results with respect to the suggested docked poses and hydrogen bond interactions to the hCA active sites.

l-(+)-Ergothioneine (**1**) showed a potent activation constant for hCA VII, whereas it displayed *K*_A_ > 100 μM for all other tested isozymes. It can adopt different docked poses for both its stereoisomers in which the acidic group of the ligand may be involved in the proton transfer from the zinc-bound water molecule to the solvent, as graphically described in Figure 2. In the first pose (Figure 2A), the carboxylic acid points towards to the zinc-bound water molecule (the shortest distance between carboxylic acid and oxygen of water molecule: 4.293 Å). Hydrogen bonds are formed with the side chains of Gln69 and Gln94, and the backbone carbonyl group of Pro203. In addition, the ligand’s sulfur atom may form an interaction with the side chain of Trp7. In the second pose (Figure 2B), the carboxyl group of the ligand forms a direct hydrogen bond to the zinc-bound water molecule. Additional hydrogen bonds are formed with the Thr202 (side chain and backbone) and Pro203. The cationic amine group of the ligand is water accessible.

**Figure 3:**
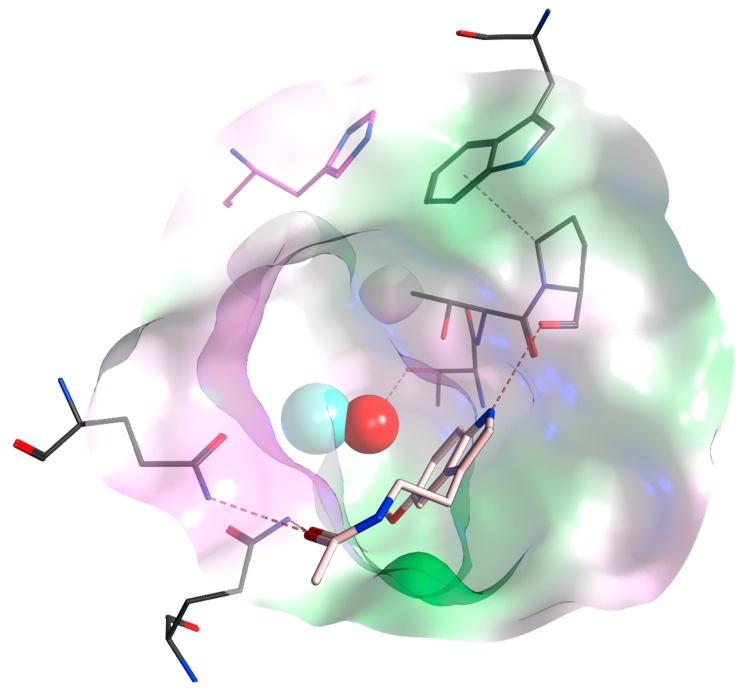
The docked pose of melatonin (**2**) in the active site of hCA VII. The proton shuttle His66 is shown in magenta.

Melatonin (**2**) formed hydrogen bonds with Gln69 and Gln94 through its carbonyl group, and with the Pro203 through its indole amine group (Figure 3). The indole ring established hydrophobic interactions with the side chain of Leu200. This docked pose placed the methoxy group of melatonin close to the zinc-bound water molecule. The hydrogen bond, that may be formed, could further assist in the transportation of the proton to the solvent.

**Figure 4:**
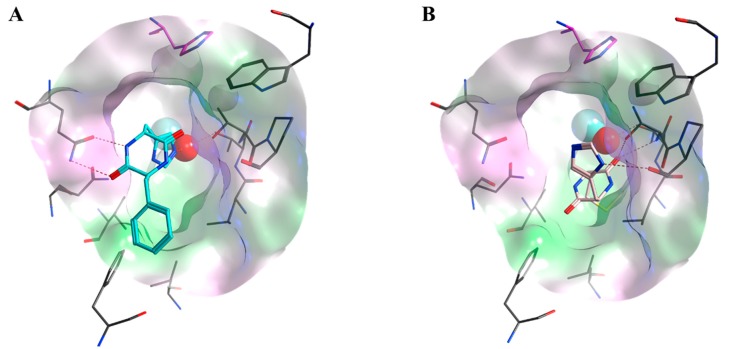
Representative docked poses of compounds **7** (**A**) and **8** (**B**) in the active site of hCA VII. The proton shuttle His66 is shown in magenta.

Compounds **3**–**6** showed similar docked poses in which the carboxylic acid moiety approached the zinc-bound water molecule in a comparable way, as observed for compound **1**. Conversely, various docked poses have been obtained for diketopiperazines **7**–**9**, in which the imidazole moiety approached the zinc-bound water molecule, or was located close to the proton shuttle His66 (Figure 4). In one docked pose for compound **7** (Figure 4A), the imidazole formed a hydrogen bond with the zinc-bound water molecule, whereas the unsubstituted phenyl group formed hydrophobic interactions with Phe133. Additional hydrogen bonds were formed with the side chain of Gln69. In another docked pose for compound **8** (Figure 4B), the imidazole ring of the ligand was pointing towards the solvent and established a hydrogen bond with the backbone of Pro203. The aliphatic tail of the ligand entered a hydrophobic pocket near Val125, Leu143 and Leu200. A hydrogen bond was also formed between the carbonyl group of the ligand and the side chain of Thr202.

Interestingly, similar docked poses to the ones obtained for hCA VII (Figure 2, Figure 3 and Figure 4) have also been observed for hCA I, II and IV despite some slight differences in their active sites as reported in Table 2. Many of the residues of hCA VII that are involved in hydrogen bonding interaction with the activators are conserved amongst hCA I, II and IV (Table 2; Trp7, Gln94 and Pro203). Only Gln69 is not conserved amongst the residues that form hydrogen bonding interactions to the ligands. As such, even though similar binding interactions may be possible, the binding strength for hCA VII is expected to be higher compared to hCA I, II and IV.

## 6. Experimental Protocols

All reactions involving air- or moisture-sensitive compounds were performed under a nitrogen atmosphere using dried glassware and syringe techniques to transfer solutions. Compounds **1**–**6** were provided by Sigma-Aldrich (Milan, Italy) and used in the biological assays without further purification (purity ≥ 98%). All other synthesized compounds (**7**–**9**) were correctly characterized by analytical and spectral data to ensure structure elucidation and purity. Column chromatography was carried out using Sigma-Aldrich^®^ silica gel (high purity grade, pore size 60 Å, 200–425 mesh particle size). Analytical thin-layer chromatography was carried out on Sigma-Aldrich^®^ silica gel on aluminum foils with fluorescent indicator. Visualization of the spots was carried out under ultra-violet (UV) irradiation (254 and 365 nm) and under development in an iodine chamber. Ashless Whatman Grade 42 (Sigma-Aldrich, Milan, Italy) filter paper was used for vacuum filtration. ^1^H-NMR (nuclear magnetic resonance) spectra were recorded on a Bruker AV300 (Bruker Biospin Corp., Billerica, MA, USA). The assignment of exchangeable protons was confirmed by the addition of D_2_O. Chemical shifts are reported in parts per million (ppm) and referenced to the characteristic non-deuterated solvent peak (2.50 ppm for hexadeuterated dimethylsulfoxide, DMSO-*d*_6_). Temperatures are reported in °C. Systematic compound names are extrapolated by ChemBioDraw Ultra^®^ 12.0 (https://chemistry.com.pk/software/free-download-chemdraw-ultra-12/) following IUPAC conventions. List of abbreviations: DMF, *N*,*N*-dimethylformamide; DCC, *N*,*N*'-dicyclohexylcarbodiimide; s.s., saturate solution; DIPEA, *N*,*N*-diisopropylethylamine; TFA, trifluoroacetic acid; DCM, dichloromethane; NMM, *N*-methylmorpholin.

*Boc(L)His-PheOMe*: To an ice cooled mixture of Boc(L)His-OH (1.0 equiv.) in DMF, DCC (1.1 equiv.), HOBt·H_2_O (1.1 equiv.), DIPEA (2.2 equiv.) and HCl·NH_2_Phe-OMe (1.0 equiv.) were added under anhydrous conditions. The mixture was stirred for 20 min and then was left to warm at room temperature (r.t.) overnight. The solvent was evaporated under reduced pressure and the solid residue was crystallized from EtOAc. All the collected organic phases were evaporated under reduced pressure. The residue was dissolved in EtOAc/1-butanol (2:1, *v*:*v*) and washed with 5% citric acid (three times), NaHCO_3_ s.s. (three times) and brine (three times). The organic phase was dried on anhydrous Na_2_SO_4_, filtered and the solvent removed under reduced pressure to give the crude product which was purified by silica gel column chromatography (gradient from CHCl_3_/MeOH = 97:3 to CHCl_3_/MeOH = 93:7, *v*:*v*) to obtain the final product in 70% yield. ^1^H-NMR (300 MHz, DMSO-*d*_6_) *δ*: 1.30 (s, 9H, Boc-His), 2.61–2.74 (m, 2H, Phe-CH_2_), 2.86–2.99 (m, 2H, His-CH_2_), 3.54 (s, 3H, Phe-OCH_3_), 4.10–4.14 (m, 1H, His-αCH), 4.43–4.47 (m, 1H, Phe-αCH), 6.69 (t, 1H, Boc-N*H*), 6.84 (d, 1H, CC*H*NH), 7.17–7.27 (m, 5H, Phe aromatic ring), 7.51 (d, 1H, amide), 8.18 (d, 1H, NC*H*NH).

*(3S,6S)-3-((1H-Imidazol-5-yl)methyl)-6-benzylpiperazine-2,5-dione* (**7**): Boc(L)His-PheOMe was deprotected with a mixture of TFA/CH_2_Cl_2_ = 1:1 (*v*:*v*) at r. t. for 1 h. The intermediate TFA salt was used for the following reaction without further purification. The so obtained product was dissolved in 0.1 M AcOH/2-butanol solution (1.5 equiv.), then NMM (1 equiv.) was added and stirred under reflux at 120 °C overnight. The solvent was removed in rotavapor, the solid residue was collected by filtration and washed with a small amount of 0.1 M AcOH/2-butanol solution, to afford the desired product in 68% yield. ^1^H-NMR (300 MHz, DMSO-*d*_6_) *δ*: 2.72–2.84 (m, 2H, Phe-CH_2_), 2.94–3.04 (m, 2H, His-CH_2_), 3.47–3.89 (m, 1H, His-αCH), 4.04–4.19 (m, 1H, Phe-αCH), 6.84 (d, 1H, CC*H*NH), 7.17–7.27 (m, 5H, Phe aromatic ring), 7.51 (d, 1H, His amide), 8.18 (d, 1H, NC*H*NH), 8.79 (d, 1H, Phe amide).

*Boc(L)His-MetOMe*: To an ice cooled mixture of Boc(L)HisOH (1.0 equiv.) in DMF, DCC (1.1 equiv.), HOBt·H_2_O (1.1 equiv.), DIPEA (2.2 equiv.) and HCl·NH_2_(L)MetOMe (1.0 equiv.) were added. The reaction mixture was stirred at 0 °C for 20 min and then it was allowed to warm at r.t. overnight. Solvent was removed under reduced pressure, the solid residue was then dissolved in EtOAc/1-butanol (2:1, *v*:*v*) and washed with 5% citric acid solution (three times), saturated solution of NaHCO_3_ (three times) and brine (three times). The combined organic phases were dried on anhydrous Na_2_SO_4_, filtered and evaporated under reduced pressure to give the desired crude product. The crude product was purified by silica gel column chromatography (eluent: CHCl_3_/MeOH = 97:3, *v*:*v*) to obtain the final product in 58% yield. ^1^H-NMR (300 MHz, DMSO-*d*_6_) δ: 1.31 (s, 9H, Boc-His), 1.85–1.92 (m, 2H, Met-CH_2_), 1.99 (s, 3H, S-CH_3_), 2.44–2.58 (m, 2H, Met-CH_2_), 2.71–2.79 (m, 2H, His-CH_2_), 3.58 (s, 3H, Met-OCH_3_), 4.08–4.14 (m, 1H, His-αCH), 4.32–4.37 (m, 1H, Met-αCH), 6.73 (d, 1H, CC*H*NH), 7.50 (d, 1H, NC*H*NH), 8.21 (d, 1H, Boc-NH), 8.28 (d, 1H, amide).

*(3S,6S)-3-((1H-Imidazol-5-yl)methyl)-6-(2-(methylthio)ethyl)piperazine-2,5-dione* (**8**): Boc(L)His-MetOMe was deprotected by a mixture of TFA/CH_2_Cl_2_ = 1:1 (*v*:*v*) at r.t. for 1 h. The intermediate TFA salt was used for the following cyclization without further purification. His-MetOMe as TFA salt was dissolved in 0.1 M AcOH/2-butanol solution (1.5 equiv.) and then NMM (1 equiv.) was added. The reaction mixture was stirred under reflux at 120 °C overnight, then it was concentrated in rotavapor and filtered; the solid was washed with a solution of 0.1 M AcOH/2-butanol and dried in rotavapor under reduced pressure. The crude product was purified by silica gel column chromatography (eluent: CHCl_3_/MeOH/H_2_O/AcOH = 70:10:10:10, *v*:*v*:*v*:*v*) to obtain the final product in 72% yield. ^1^H-NMR (300 MHz, DMSO-*d*_6_) δ: 1.99 (s, 3H, S-CH_3_), 2.25–2.32 (m, 2H, Met-CH_2_), 2.86–2.93 (m, 2H, Met-CH_2_), 3.79–3.86 (m, 1H, His-αCH), 4.01–4.05 (m, 1H, Met-αCH), 6.76 (d, 1H, CC*H*NH), 7.51 (d, 1H, NC*H*NH), 8.06 (d, 1H, His-amide), 8.14 (d, 1H, Met-amide).

*Boc(L)His-ProOMe*: To an ice cooled mixture of Boc(L)HisOH (1.0 equiv.) in DMF, DCC (1.1 equiv.), HOBt·H_2_O (1.1 equiv.), DIPEA (2.2 equiv.) and HCl·NH-ProOMe (1.0 equiv.) were added. The reaction mixture was stirred at 0 °C for 20 min and then it was allowed to warm at r.t. overnight. The reaction mixture was evaporated in rotavapor. The residue was dissolved in EtOAc/1-butanol (2:1, *v*:*v*) and washed with three portions of 5% citric acid solution (three times), NaHCO_3_ s.s. (three times) and brine (three times). The combined organic phases were dried on anhydrous Na_2_SO_4_, filtered and evaporated under reduced pressure to give the crude product which was purified by silica gel column chromatography (eluent: CHCl_3_/MeOH = 97:3, *v*:*v*) to obtain the final product in 77% yield. ^1^H-NMR (300 MHz, DMSO-*d*_6_) *δ*: 1.30 (s, 9H, Boc-His), 1.82–1.91 (m, 3H, Pro-CH_2_), 2.14–2.23 (m, 1H, Pro-CH_2_), 2.67–2.74 (m, 3H, His-CH_2_ + Pro-CH), 3.56–3.62 (m, 4H, 1H-Pro-CH + 3H Pro-OCH_3_), 4.29 (m, 1H, Pro-αCH), 4.32 (m, 1H, His-αCH), 6.80 (d, 1H, CC*H*NH), 7.13 (d, 1H, Boc-N*H*), 7.52 (d, 1H, NC*H*NH).

*(3S,8aS)-3-((1H-Imidazol-5-yl)methyl)hexahydropyrrolo*[1,2-a]*pyrazine-1,4-dione* (**9**): Boc(L)His-ProOMe was deprotected with a mixture of TFA/CH_2_Cl_2_ = 1:1 (*v*:*v*) at r.t. for 1 h. The intermediate TFA salt was used for cyclization reaction without further purification. (L)His-ProOMe as TFA salt was dissolved in 0.1 M AcOH/2-butanol solution (1.5 equiv.) and NMM (1 equiv.) was added. The resulting solution was stirred under reflux at 120 °C overnight. The solvent was concentrated in rotavapor, followed by trituration with diethyl ether. The crude product was purified by silica gel column chromatography (eluent: CHCl_3_/MeOH/H_2_O/AcOH = 75:27:5:0.5, *v*:*v*:*v*:*v*) to obtain the final product in 71% yield. ^1^H-NMR (300 MHz, DMSO-*d*_6_) δ: 1.82–1.91 (m, 2H, Pro-CH_2_), 2.10 (m, 1H, Pro-CH_2_), 2.71–2.70 (m, 3H, His-CH_2_ + Pro-CH), 3.60 (m, 1H, 1H-Pro-CH), 4.17–4.10 (m, 2H, Pro-αCH + His-αCH), 4.32 (m, 1H, His-αCH), 6.87 (d, 1H, CC*H*NH), 7.56 (d, 1H, NC*H*NH), 8.06 (d, 1H, amide).

### 6.1. Carbonic Anhydrase Assays

A stopped-flow method has been used for assaying the CA-catalyzed CO_2_ hydration activity with Phenol red as an indicator, working at the absorbance maximum of 557 nm, following the initial rates of the CA-catalyzed CO_2_ hydration reaction for 10–100 s [38]. For each activator, at least six traces of the initial 5–10% of the reaction have been used for assessing the initial velocity. The uncatalyzed rates were measured in the same manner and subtracted from the total observed rates. Stock solutions of activator (0.1 mM) were done in distilled-deionized water and successive dilutions up to 0.1 nM were then performed with the assay buffer. The activation constant (*K*_A_), defined similarly to the inhibition constant *K*_I_, was calculated by the classical Michaelis-Menten equation (Equation (1)) fitted by non-linear least squares by using PRISM 3:

v = v_max_/{1 + *K*_M_/[S] (1 + [A]_f_/*K*_A_)} (1)

where [A]_f_ is the free concentration of activator.

Working at substrate concentrations markedly lower than *K*_M_ ([S] << *K*_M_), and keeping in mind that [A]_f_ can be expressed as the total concentration of the activator ([A]_t_) and enzyme ([E]_t_), the obtained competitive steady-state equation for measuring the activation constant is provided by Equation (2):

v = v_0_·*K*_A_/{*K*_A_ + ([A]_t_ − 0.5 {([A]_t_ + [E]_t_ + *K*_A_) − ([A]_t_ + [E]_t_ + *K*_A_)^2^ − 4[A]_t_·[E]t)^1/2^}} (2)

where v_0_ is the initial velocity of the enzymatic reaction in the absence of activator. All carbonic anhydrase isoforms used in these experiments were obtained in-house as purified recombinant proteins [39,40,41,42,43,44].

### 6.2. Molecular Modelling Studies

#### 6.2.1. Preparation of Files for Docking Studies

Crystal structures of hCA I (pdb: 3lxe, 1.9 Å, in complex with topiramate), II (pdb: 4e3d, 1.6 Å, in complex with 2,5-dihydroxybenzoic acid), IV (pdb: 3fw3, 1.72 Å, in complex with dorzolamide) and VII (pdb: 3mdz, 2.32 Å, in complex with ethoxzolamide) were obtained from the Brookhaven Protein Data Bank. All ligands (topiramate, 2,5-dihydroxybenzoic acid, ethoxzolamide and dorzolamide) and the zinc-bound water molecule of hCA II were retained and all other non-protein atoms were deleted. Hydrogen atoms were added with the “protonate 3D” tool, and subsequently a steepest-descent energy minimization was performed using the AMBER12:EHT force field (MOE software package, version 2015.10, chemical computing group, Inc., Montreal, QC, Canada). The four protein structures were superposed on the backbone atoms of hCA I (Cα atoms, RMSD: 1.395 Å, for 236 residues). The coordinates of the hCA II zinc-bound water molecule were copied into the other hCA structures.

The molecular structures of the activators were prepared with the MOE software package. All stereoisomers were generated. All strong bases were protonated and all strong acids were deprotonated. Subsequently, the ligand structures were energy minimized (MMFF94x force field) and the ligands were saved as multi-mol2 files.

#### 6.2.2. Docking Studies

Docking calculations were performed with the GOLD software package (v5.4, CCDC, Cambridge, UK) using the ChemScore scoring function (50 dockings) and default settings [45,46]. The binding pocket was defined as within 16 Å around a centroid (*x*: −18.459, *y*: 35.3775, *z*: 43.564; OAR atom of topiramate, 3lxe structure) as previously reported [45,46].

## 7. Conclusions

Due to the significantly diminished activity or expression of specific isoforms of carbonic anhydrases in the brain of patients suffering from ageing or Alzheimer’s diseases, we proposed novel nitrogen containing chemotypes incorporating five- or six-membered cycles able to elicit the proton shuttle, and thus to activate the catalytic activity of CA. Natural and synthetic compounds were investigated against four isoforms of CA usually involved in CNS development and functioning. We obtained promising results in terms of activation activity (up to nanomolar range) and isoform selectivity (especially towards hCA VII).

## Figures and Tables

**Table 1:** Experimental activation constants (*K*_A_s) of hCA I, hCA II, hCA VII (cytosolic isozymes) and hCA IV (membrane-associated isoform) for compounds **1**–**9**. Histamine (Hst) was used as reference compound.

Compound	Structure	*K*_A_ (μM) *			
		hCA I	hCA II	hCA IV	hCA VII
**1**	l-(+)-Ergothioneine	>100	>100	>100	0.82
**2**	Melatonin	>100	>100	53.4	0.12
**3**	4-Imidazole-acrylic acid	21.3	>100	64.7	3.46
**4**	1*H*-Indazole-3-COOH	32.4	>100	78.4	5.23
**5**	TIC^.^HCl	23.1	>100	73.5	3.61
**6**	Spinacine	7.21	>100	69.7	15.7
**7**	c(HisPhe)	16.3	>100	80.5	15.1
**8**	c(HisMet)	8.39	>100	77.0	21.6
**9**	c(HisPro)	7.92	>100	70.1	34.7
**Hst**	Histamine	2.10	125	36.9	25.3

* Mean from three different determinations (errors in the range of 5–10% of the reported values, data not shown).

**Table 2:** Comparison of the active sites among the four selected hCA isoforms.

hCA I (3lxe)	hCA II (4e3d)	hCA IV (3fw3)	hCA VII (3mdz)
Trp5 *	Trp5	Trp5	Trp7
Val62	Asn62	Asn62	Asn64
His64	His64	His64	His66
His67	Asn67	Met67	Gln69
Phe91	Ile91	Lys91	Lys93
Gln92	Gln92	Gln92	Gln94
His94	His94	His94	His96
His96	His96	His96	His98
His119	His119	His119	His121
Ala121	Val121	Val121	Val123
Leu131	Phe131 ***	--Val131--	Phe133
Ala132	Gly132	--Lys132-- **	Gly134
Ala135	Val135	--Gln135--	Ala137
Leu141	Leu141	Ile141	Leu143
Val143	Val143	Val143	Val145
Leu198	Leu198	Leu198	Leu200
Thr199	Thr199	Thr199	Thr201
His200	Thr200	Thr200	Thr202
Pro201	Pro201	Pro201	Pro203
Pro202	Pro202	Thr202 **	Pro205
Tyr204	Leu204	Asp204	Ser206
Val207	Val207	Val207	Val209
Trp209	Trp209	Trp209	Trp211

* conserved but different rotamer compared to the rest; ** points away from ligand (or will point away from hCA IV ligand); *** the region near Phe131 (hCA II) is not resolved in the hCA IV (3fw3) structure.
